# Temporal Variability of Zooplankton (2000-2013) in the Levantine Sea: Significant Changes Associated to the 2005-2010 EMT-*like* Event?

**DOI:** 10.1371/journal.pone.0158484

**Published:** 2016-07-26

**Authors:** Anthony Ouba, Marie Abboud-Abi Saab, Lars Stemmann

**Affiliations:** 1 National Council for Scientific Research, National Center for Marine Sciences, P.O. Box 534, Batroun, Lebanon; 2 CNRS, UMR 7093, LOV, Observatoire Océanologique, F-06230, Villefranche-sur-Mer, France; 3 Sorbonne Universités, UPMC Université Paris 06, CNRS, Laboratoire d’Océanographie de Villefranche (LOV) UMR7093, Observatoire Océanologique, 06230, Villefranche-sur-Mer, France; Technical University of Denmark, DENMARK

## Abstract

In this study, we investigated, for the first time, the potential impact of environmental changes on zooplankton abundance over a fourteen year period (2000–2013) at an offshore station in the Eastern Mediterranean Sea (the Levantine basin, offshore Lebanon). Samples were collected monthly and analyzed using the semi-automated system ZooScan. Salinity, temperature and phytoplankton abundance (nano and microphytoplankton) were also measured. Results show no significant temporal trend in sea surface temperature over the years. Between 2005–2010, salinity in the upper layer (0–80 m) of the Levantine basin increased (~0.3°C). During this 5 year period, total zooplankton abundance significantly increased. These modifications were concomitant to the activation of Aegean Sea as a source of dense water formation as part of the “Eastern Mediterranean Transient-*like*” event. The results of the present study suggested that zooplankton benefited from enhanced phytoplankton production during the mixing years of the event. Changes in the phenology of some taxa were observed accordingly with a predominantly advanced peak of zooplankton abundance. In conclusion, long-term changes in zooplankton abundance were related to the Levantine thermohaline circulation rather than sea surface warming. Sampling must be maintained to assess the impact of long-term climate change on zooplankton communities.

## Introduction

Zooplankton communities are crucial components of marine ecosystems due to their central role in marine trophic food webs and their impact on carbon cycling [1]. Zooplankton communities are sensitive to climate change [2] and vulnerable to changes in the hydrography [3]. They are able to respond rapidly to any ecosystem variability [4, 5]. Long-term plankton time-series play an essential role in detecting such environmental changes [6–8]. They are suitable tools in capturing the modes of population, the community structure and the changes at different temporal scales. Compared to the Pacific [9] and Atlantic Oceans [10–12], fewer zooplankton long-time series are available for the Mediterranean Sea area [13]. There are seven ongoing time-series, from which four are concentrated in the NW Mediterranean [14–19], two are carried out in the Adriatic Sea [20, 21] and one in the Aegean Sea [22, 23]. Previous studies in the Western Mediterranean sea showed decadal changes in zooplankton key groups and community structure associated with modifications in atmospheric conditions in the 1980s [14, 24], early 1990s and 2000s [24, 25] in the Ligurian basin and in the mid-90s in the Balearic Sea [26]. In the coastal Aegean Sea, zooplankton decadal changes were probably triggered by changes of anthropogenic origin rather than change in climatic forcing [23].

Since the 1990s, drastic changes in the circulation of deep water masses at the Eastern Mediterranean (EMed) are known as the Eastern Mediterranean Transient (EMT). The EMT is a shift of deep waters from its usual southern Adriatic source to a new Aegean source due to a combination of exceptional meteorological and hydrological factors [27–36]. This event changes the circulation from the deep layers up to the euphotic zone causing a modification in water characteristics [37]. Recently, renewed interests on the functioning of EMed thermohaline circulation as an anti-correlated oscillation between the Aegean and the Adriatic seas were revived [37]. It was manifested almost every decade. Therefore, the EMT event was represented by alternation of intense-non intense cycles of Dense Water Formation (DWF) due to internal driving mechanism [37, 38]. Starting 2004–2005, the Aegean Sea became an active dense water source area. It has been detected by the salinity increase in the upper, intermediate and deep layers of the EMed [38–40]. After 2010, salinity returned back to its pre-2005 condition [38, 39]. Unlike the 1990s event, Krokos et al. [38] defined this episode as “EMT-*like*” event due to an internal mechanism without any intervention of any extreme atmospheric forcing.

The EMT onset affected the distribution of zooplankton abundance and composition between the northwestern and the eastern areas of the Ionian Sea [41]. For example, new copepod species were observed in the north Adriatic Sea as well as a significant rise or decline of several key species [42]. Total copepod abundance increased in the eastern Saronikos Gulf after the EMT onset [22]. A remarkable increase in zooplankton abundance and an appearance of new copepod species with the dominance of the calanoïd were also detected in the deep Ieraptera basin of the Levantine Sea [43]. The existing data are still limited and based on episodic cruises in the open Levantine Sea [41, 43–47]. In the Lebanese waters, zooplankton studies were restricted to analysis of seasonal variation in diversity [48–56]. However, a potential synchronicity between the EMT-*like* event and the zooplankton community changes remains unknown in the Levantine Basin.

Here we report results from a 14-year long time-series in the Levantine basin during which consistent sampling strategy for physical and biological key variables was applied. Therefore, the aims of the present work are (1) to provide an overview on the interannual variability of the zooplankton abundance, (2) to identify what underlying mechanism interacts probably the most with the zooplankton community in the Lebanese waters and finally (3) to assess the zooplankton variability possibly triggered by the EMT-*like* event that occurred between 2005 and 2010.

## Methods

### Sampling site

**Fig 1:**
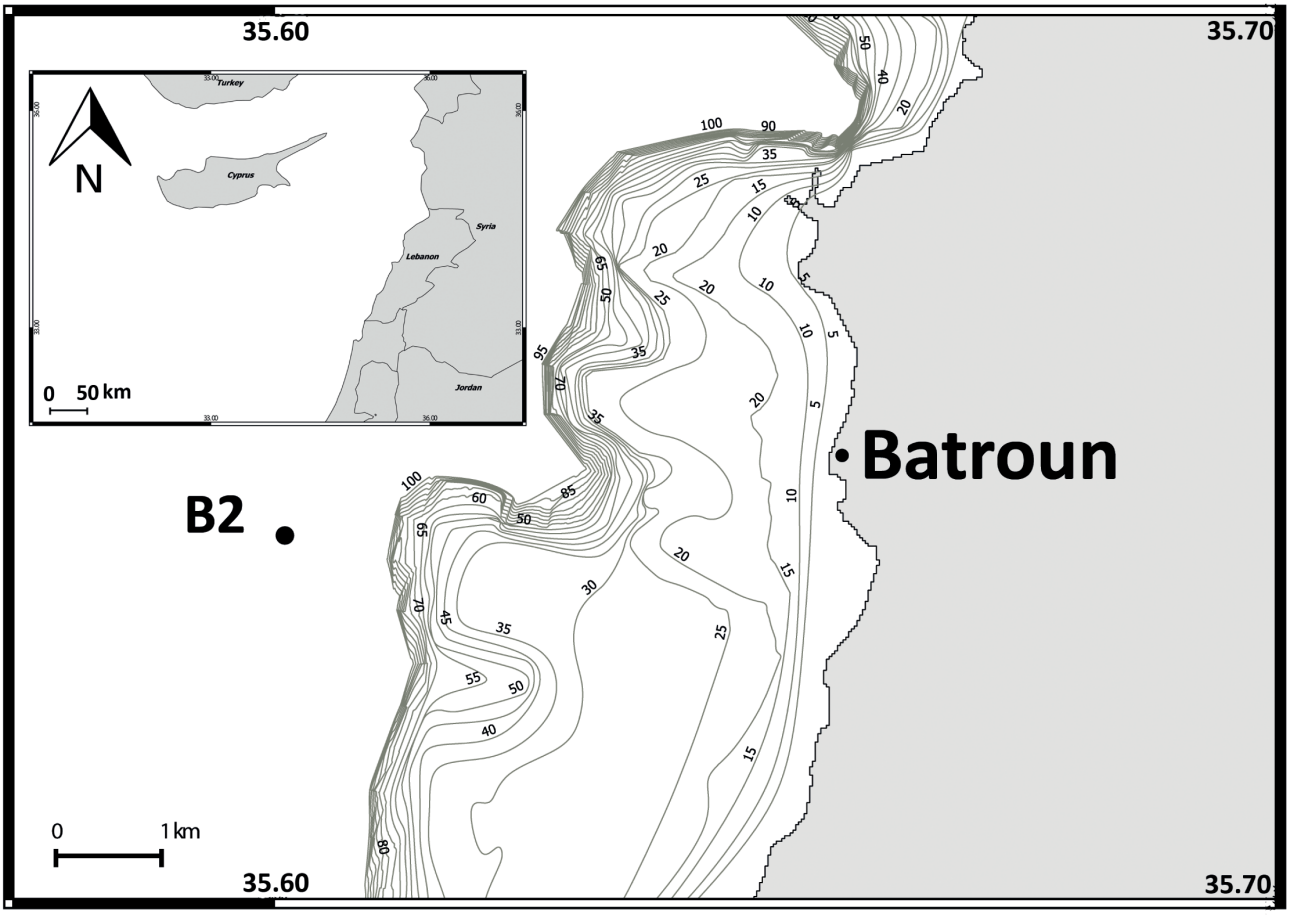
Location of Lebanon in the Levantine Basin, and the sampling site (Point B2) in the Lebanese waters.

The monitored station, B2 (N 34°14,856; E 35°36,067) is located offshore Batroun city (north Lebanon). It is almost 4 miles offshore over a bottom depth of 500 m (Fig 1). Despite this fact, it represents the open sea conditions due to the narrow continental shelf (less than two miles) and the rapid increase in depth. This station is a part of the long-term research program (2000 to 2013) carried out in this area by the Lebanese National Center for Marine Sciences (NCMS). Water samples were collected monthly during daytime (between 07:00 and 11:00). Sampling frequency was homogeneous over the time. In case of sampling failure (due to technical problems or storms), the campaigns were compensated by additional samplings 1 or 2 weeks later. No specific permissions were required for this location because it is a public area to which we have full access due to its proximity to our institute. Finally, these field studies did not involve endangered or protected species.

### Environmental and phytoplankton data

Water temperature (°C) and salinity were measured at five different depths (0, 20, 40, 60 and 80 m) using sequentially the same Niskin bottle. Depth average values (0-80m depth) were used for the analysis. At each depth, the temperature was recorded with a reversing thermometer (Richter & Wiese type, 0.05°C precision) and the salinity was determined with a Beckman induction salinometer (model RS7-C with a precision of 0.001). Water samples for phytoplankton cells counts were also taken from the Niskin bottle at 0, 20, 40 and 60m depth and they were immediately preserved with lugol’s solution (0.5% as a final concentration). Species were counted using Utermöhl’s sedimentation method [57]. No inverted filtration was carried out for the concentration. Instead, a volume of 100 ml of each sample was placed for 48h in a 25 mm diameter sedimentation chamber. Then, the base of the chamber was examined with a Wild M 40 phase-contrast inverted microscope. Counts were performed with 200x magnifications for microphytoplankton (> 20 μm) and 400x magnifications for smaller cells (nanoplankton).

### Zooplankton collection and analysis

Zooplankton samples were also collected monthly from January 2000 till December 2013 at the point B2. Vertical hauls were made through the water column, from a depth of 60 m to the surface with a 40 cm opening diameter net of 52 μm mesh size. The sampled volume of ~7.8 m^3^ was estimated from the vertical towed height and the net opening surface (0.13 m^2^). The net was gently rinsed and the catches were immediately transferred and preserved in formaldehyde (4% as a final concentration) buffered with borax (Sodium Borate) for subsequent analysis.

Samples (165 in total) were split each into two halves with the Folsom Divider. Only one half of each zooplankton sample was analyzed and digitized with ZooScan (hydroptic v3 window 7), an imaging system developed in the Laboratory of Oceanography of Villefranche (LOV) [58]. The other half was stored for long-term archiving. The ZooScan methodology is based on pattern recognition of zooplankton images and it enables the count, the measure of the size and the classification of the organisms in order to provide their numerical abundances. Each sample of this time-series was sieved through a 150 μm mesh size to remove the smaller organisms (< 150 μm) that cannot be detected with ZooScan [59]. Then, each fraction was diluted with a Motoda box [60] to yield an average of ~1000 objects in the scanning tray, in order to permit an easy manual separation of organisms. Sub-samples were digitized at 4800 dpi resolution (each pixel was equivalent to 5.29 μm^2^). After the scanning step, 165 raw images of digital data set in total were obtained for the entire time-series (one image per sample). Image processing and the image acquisition of the data were done using Zooscan, an image analysis software. Automatic classification by supervised-learning was performed by the “Plankton Identifier” (PkId) [61], based on Tanagra data mining software [62], and validation of the classification was done manually. The learning set used for the automatic classification of object in different categories was the same one built for the zooplankton time-series in Villefranche-sur-Mer [25], in addition to some modifications in order to increase the accuracy of sorting. The classifier was composed of 36 categories (31 zooplankton of different taxonomic groups or genera and 5 non-zooplankton including detritus and artifacts). For details on the methodology of the Zooscan integrated system, refer to Gorsky et al. [58]. Organisms were further grouped into nineteen zooplankton categories which were unambiguously identified in all samples. Copepods were identified at genus level (*Oithona* spp, *Calanus* spp, *Corycaeus* spp, *Temora* spp and *Oncaea* spp) except the Harpacticoïds. Two genera of the cladocerans were identified: *Penilia* and *Evadne* spp. As for larvae, four groups were identified: euphausiids, echinoderms, cirripedia and nauplii, whereas the remaining groups were identified at higher taxonomic levels (appendicularians, annelids, chaetognaths, gastropods, jellyfish, thaliacea, siphonophores, ostracods, eggs, pteropods and others). The taxonomic composition of zooplankton in the Lebanese waters was described in details by Lakkis [48].

### Data analyses

Graphs and analysis were performed using R Development Core Team [63] and Ocean Data Viewer (ODV). First, regularization of all time-series were made with “R” using the linear method with a delta t = 30 days. The annual and the monthly mean of environmental variables were calculated using the ODV program. The rank based non-parametric Mann-Kendall test was used for detecting trends in this time-series data. To identify significant shifts in the time-series of hydrographic variables and total zooplankton abundance, we used the sequential regime-shift detection method (STARS, www.beringclimate.noaa.gov) [64, 65] as already used by Vandromme et al. [25] and Möllmann et al. [66]. The cut-off length, which determines the minimum length of a regime, was set at 23 month for the analyses of the zooplankton time-series and 30 for the salinity time-series. The Wilcoxon rank sum test (W) was used to test the differences in zooplankton groups and genera abundances and in hydrographic parameters between the two defined periods. The significance level for all the tests was always set to p = 0.05.

## Results

### Long-term environmental variability

**Fig 2:**
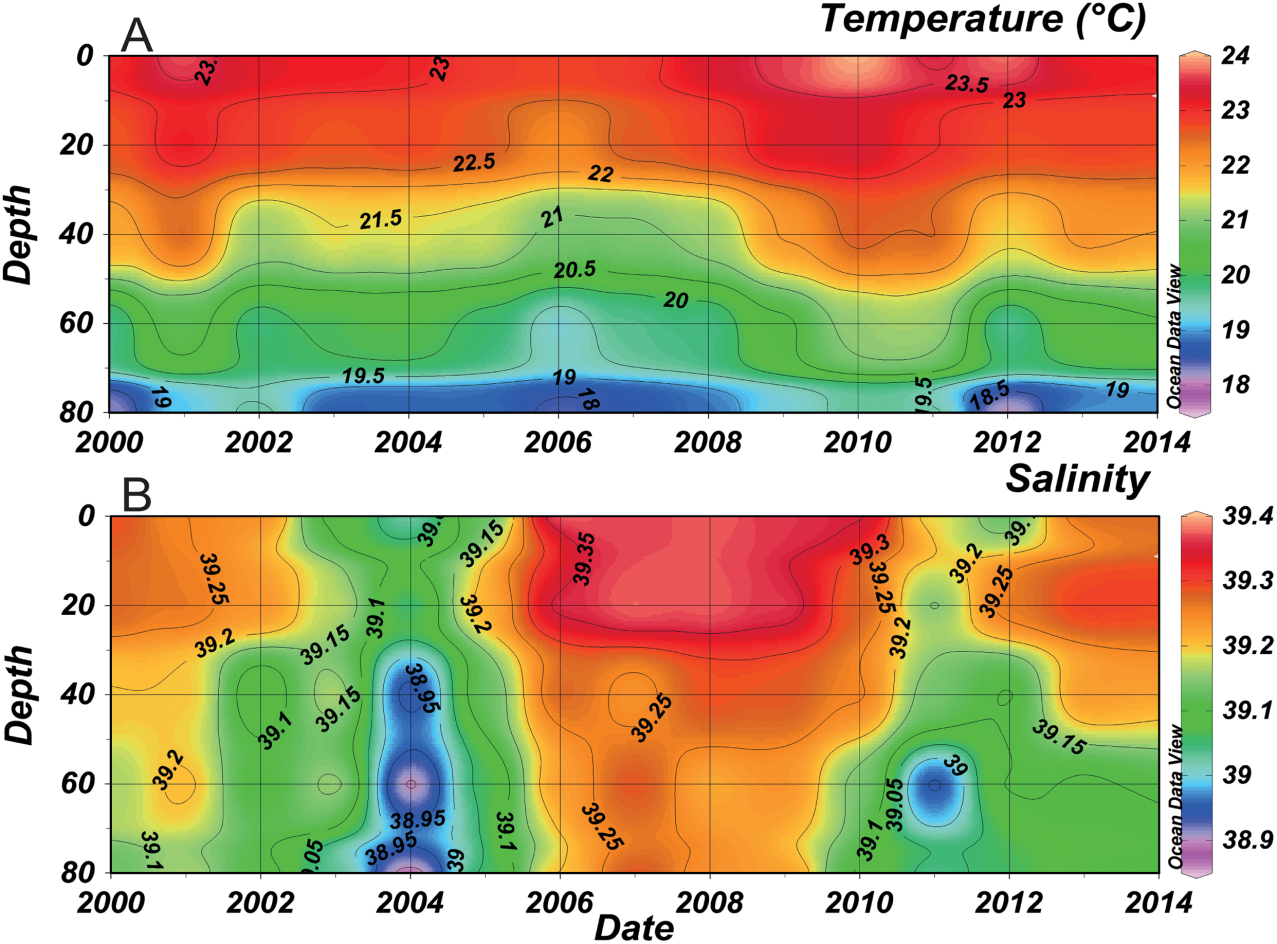
Temporal evolution of mean annual temperature (A) and salinity (B) along the water column at B2 between 2000 and 2013 (refer to S1 Table for more details on temperature and salinity).

Temperature and salinity decreased relatively with the increasing depth (Fig 2). Annual mean temperature showed a fluctuation between 23 and 23.5°C on the surface and reached 19°C at 80 m depth (Fig 2A). An increase in mean temperature occurred in 2001, 2010 and 2011 in the whole water column, whereas it decreased from 2004 till 2008. Lowest salinity values were recorded between 2003 and 2005 (39.05 on the surface and 38.85 at 80 m depth) (Fig 2B). In early 2005, an increase of almost 0.3 in water column salinity was witnessed, reaching 39.35 at the surface and 39.2 at a depth of 80 m. These high salinity values were observed till 2010 and then decreased during 2011 and 2012 (39.15 in the surface and 39 at 80 m depth). No significant temporal trend was evident in both temperature and salinity over this time-series.

**Fig 3:**
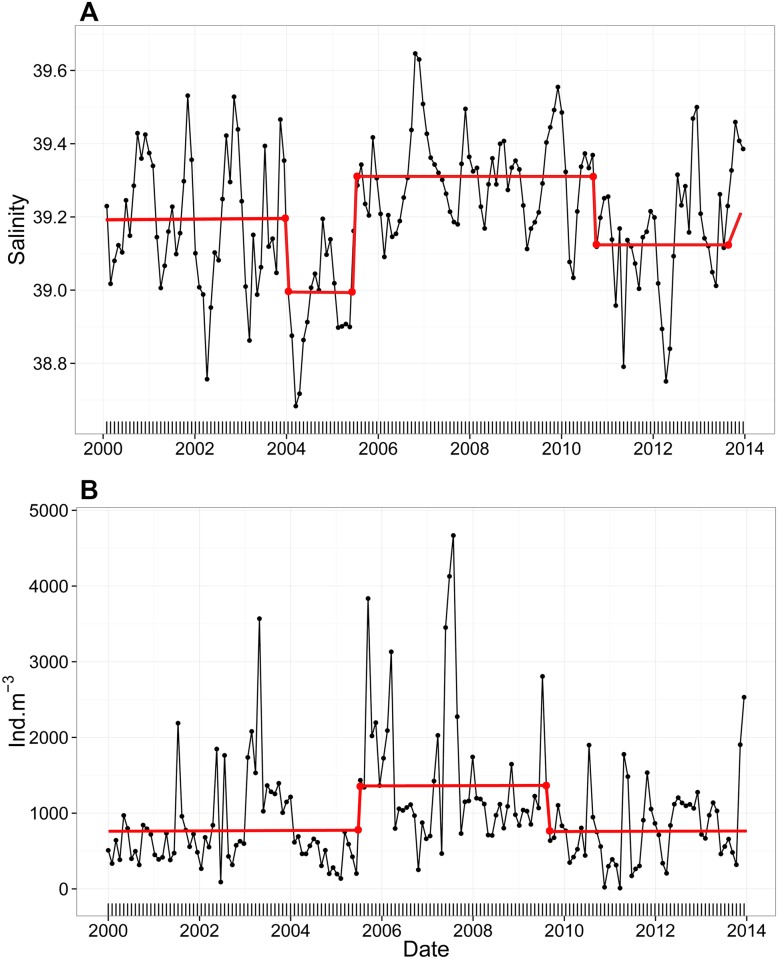
Monthly time-series of the (A) mean salinity values and (B) total zooplankton abundance in the water column at B2 between 2000 and 2013 (refer to S2 Table for more details on total zooplankton). The red line corresponds to the STARS shift detection method [64] that detects significant changes in the mean (α = 0.05).

Using STARS method, we noticed that the shift to higher salinity values was significant from June 2006 till October 2010 and opposite changes, a significant shift to lower values, occurred in 2004-mid 2005 and starting mid-2010 (Fig 3A). Total zooplankton also followed the corresponding shift (Fig 3B) and experienced a significant increase in the abundance between July 2005 and March 2010 (from 840.5 ± 577.9 to 1211.8 ± 917.9 ind.m^-3^). According to that, two periods were identified in the present study: the first one as a saline period (SP) corresponding to 2005–2010 and the second one as a non-saline period (NSP) corresponding to 2000–2004 and 2011–2013.

**Fig 4:**
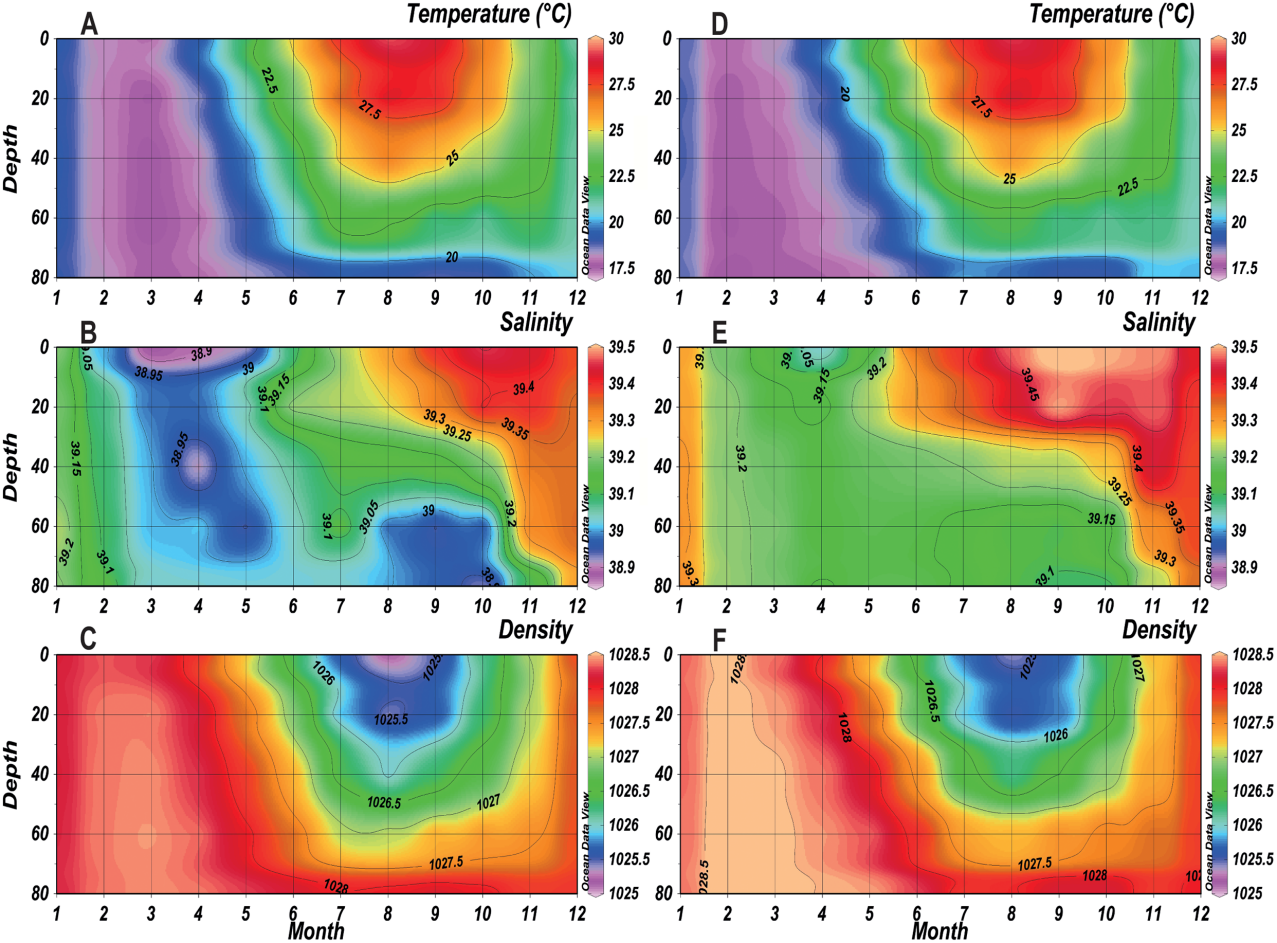
Monthly mean temperature, salinity and density of the NSP (A, B, C respectively) and SP (D, E, F respectively) in the water column at B2.

A comparison of annual cycles in physical parameters (temperature, salinity and density) between the two periods is shown in Fig 4. Temperature displayed a clear seasonality characterized by 1) a period with a homogeneous mixed water column from January to April where temperature was below 19°C and 2) a period with a stratified water column for the remaining year where the temperature was higher than 27°C at the sea surface in both saline and non-saline periods. During winter (January-March), the observed temperature during the SP (Fig 4D) was lower, minimum values occurred earlier (minimum of <17.5°C in February) and were more homogeneous along the water column than the NSP (minimum of 17.5°C in March) (Fig 4A). During summer (July-September), the thermocline during the SP seemed to be shallower and less stratified (above 60 m depth in July) than the NSP (below 60 m depth).

As for the salinity, a strong stratification was observed through the water column in both periods with a clear salinity rise of 0.2–0.3 for the SP (Fig 4E). A homogeneous water column was observed from February to May with salinity ranging between 38.9 and 39 for the NSP (Fig 4B) and between 39.05 and 39.2 for the SP. By May, the salinity started to increase at the surface toward the depth at both periods. Salinity peaked at the surface between September and November and reached 39.45 during the NSP, whereas it reached 39.5 between August and November during the SP.

Regarding seawater density, vertical gradients were minimum in February during winter of the SP (> 1028 Kg.m^-3^), one month earlier than the NSP (Fig 4C and 4F). However during summer, a weaker and narrower density gradient was evident during the SP. Values of 1026 Kg.m^-3^ ranged between 0 and 30 m in July-September during the SP, while they ranged between 0 and 40 m during August of the NSP. Also, values of 1026.5 Kg.m^-3^ were below 40 m during August of the SP and during July-September of the NSP.

### Seasonal variability of phytoplankton

**Fig 5:**
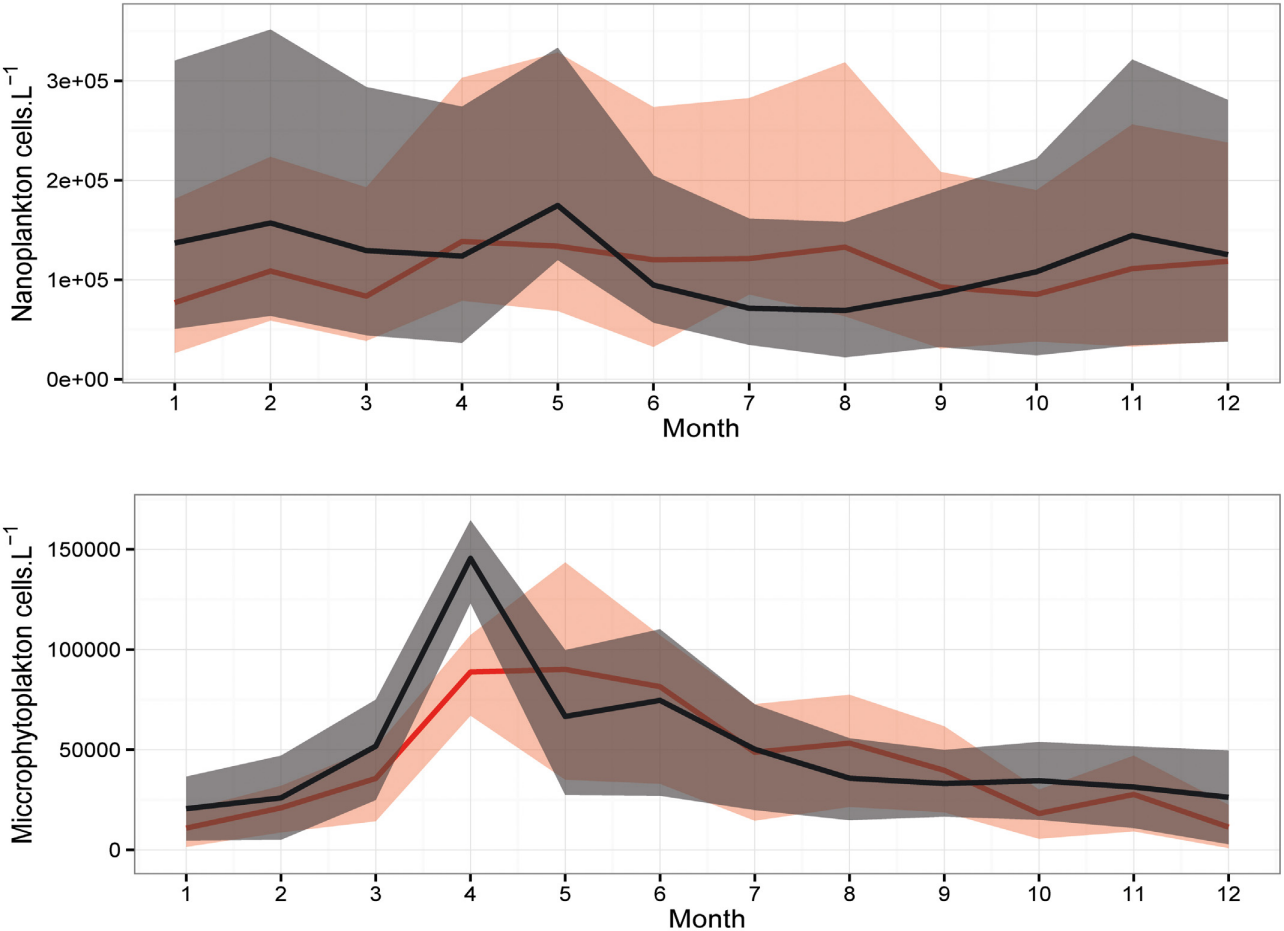
Annual cycle of mean abundances of the (A) Nanoplankton and (B) Microphytoplankton during SP (black) and NSP (red) with percentiles Q1 and Q3 (shaded area) at B2.

The comparison in the mean annual cycle of the phytoplankton populations (Fig 5) shows the difference between the two identified periods. Regarding the nanoplankton, a significant difference (p < 0.05) was only detected in the winter between the SP and the NSP. However, a clear difference in the phenology of the two periods was detected. During the NSP, the nanoplankton population did not present a clear annual cycle in their abundances (Fig 5A). It increased in the spring-early summer and mean values reached 1.4.10^5^ cells.L^-1^. In contrast, during the winter, spring and autumn of the SP, mean abundances were higher than the NSP and showed peaks in February (1.6.10^5^ cells.L^-1^), May (1.7.10^5^ cells.L^-1^) and November (1.4.10^5^ cells.L^-1^).

Regarding the microphytoplankton (diatoms and dinoflagellates), a significant difference was detected between the SP and the NSP (p < 0.05). A clear seasonality was also noticed in their annual cycle for both periods with a difference in the timing of the peaks (Fig 5B). Mean abundances reached a maximum of 9.10^4^ cells.L^-1^ in May of the NSP whereas in the SP the mean values peaked one month earlier (1.45.10^5^ cells.L^-1^ in April).

### Interannual zooplankton variability

A total of 25 zooplankton taxa of different taxonomic levels were identified in the sampling site. Descriptive statistics (mean and standard deviation) of taxa abundances for each period are showed in Table 1. The difference in abundances between the two periods was significant. The test results confirmed a significant increase (p < 0.05) in abundances between the two periods for total zooplankton (44%), total copepods (47%) including the *Calanus* spp, *Acartia* spp, *Oithona* spp and *Corycaeus* spp, annelids, pteropods, gastropods, siphonophores, chaetognaths and eggs (all increasing during the SP).

**Table 1:** Descriptive statistics (Average and Standard Deviation) of the identified zooplankton in the sampling site and the Wilcoxon test showing the difference betweeen the SP and NSP in p-value order.

Groups	NSP Average ± SD (ind.m^-3^)	SP Average ± SD (ind.m^-3^)	*p*-value
**Others**	5.6 ± 5.7	5.1 ± 4.9	0.916
**Euphausiacea**	2.5 ± 8.9	1.3 ± 2	0.813
***Evadne* spp**	13.7 ± 35.5	27.7 ± 80.8	0.721
**Jellyfich**	11.2 ± 13.4	11.8 ± 14.1	0.63
**Appendicularians**	53.3 ± 67.2	55.9 ± 61.4	0.598
**Ostracods**	6.9 ± 7.1	6.6 ± 7.2	0.589
**Cirripedia**	5.1 ± 11.5	13.2 ± 53.9	0.548
**Thaliacae**	4.7 ± 9.2	12.7 ± 43.8	0.494
***Oncaea* spp**	16.9 ± 15.8	22 ± 23.8	0.359
***Penilia* sp**	32.2 ± 16.6	1.6 ± 6.3	0.344
***Temora* spp**	23.4 ± 41.2	38.9 ± 72.9	0.336
**Echinoderm**	2.6 ± 6.1	3.4 ± 6.2	0.184
**Harpacticoïds**	15.5 ± 16.3	23 ± 28.4	0.181
**Nauplii**	4 ± 3.8	3.7 ± 5	0.114
***Calanus* spp**	31.7 ± 29.4	42.9 ± 39.8	**0.022**
**Annelids**	18.3 ± 19.3	30.1 ± 37.1	**0.018**
**Pteropods**	5.4 ± 13.7	5.5 ± 7.5	**0.016**
***Acartia* spp**	0.8 ± 1.5	2.5 ± 4.1	**0.015**
***Oithona* spp**	111 ± 96.2	168.1 ± 195.6	**0.014**
**Eggs**	5.4± 7.4	8.1 ± 7.8	**0.011**
***Corycaeus* spp.**	28.8 ± 29.8	42.7 ± 40.9	**0.004**
**Total zooplankton**	840.5 ± 577.9	1211.8 ± 917.9	**0.002**
**Total copepods**	684.2 ± 486.3	1011.1 ± 800.6	**0.001**
**Chaetognaths**	18.2 ± 21.2	28.8 ± 30.4	**0.0006**
**Gastropods**	11.9 ± 27.1	14.2 ± 35.4	**0.0001**
**Siphonophores**	5 ± 4.7	10.5 ± 7.5	**3.251.10**^**−7**^

**Fig 6:**
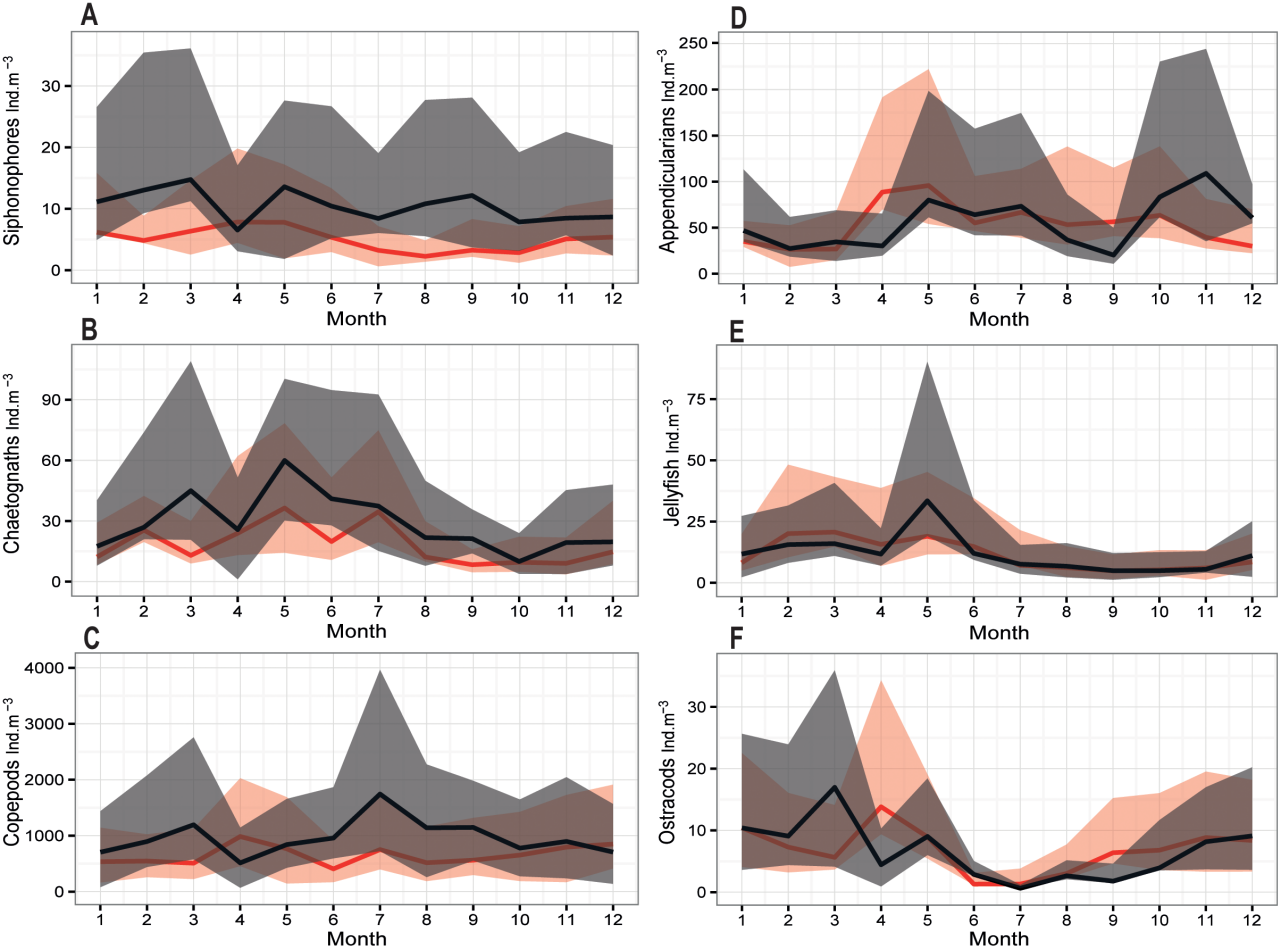
Annual cycle of mean abundances of the (A) Siphonophores, (B) Chaetognaths, (C) Copepods, (D) Appendicularians, (E) Jellyfish and (F) Ostracods during SP (black) and NSP (red) with percentiles Q1 and Q3 (shaded area) at B2.

A noticeable feature in the mean annual cycle analysis of some selected zooplankton groups is the difference in the two identified periods (Fig 6). Mean values of the siphonophores (Fig 6A), chaetognaths (Fig 6B) and copepods (Fig 6C) were clearly higher along the years of the SP, with few exceptions. During SP, siphonophores and copepods showed slight lower abundances than the NSP in April. Copepods also displayed lower values in December during the SP. Appendicularians (Fig 6D), jellyfish (Fig 6E) and ostracods (Fig 6F) did not show any significant difference in their annual cycle between the two periods. In addition, changes in the phenology have occurred for some groups. Siphonophores, copepods and ostracods peaked in March during the SP which is one month earlier than the NSP (April). A two months shift was observed for the chaetognaths (May). It is noteworthy that other peaks appeared in other seasons for siphonophores, appendicularians and copepods.

**Fig 7:**
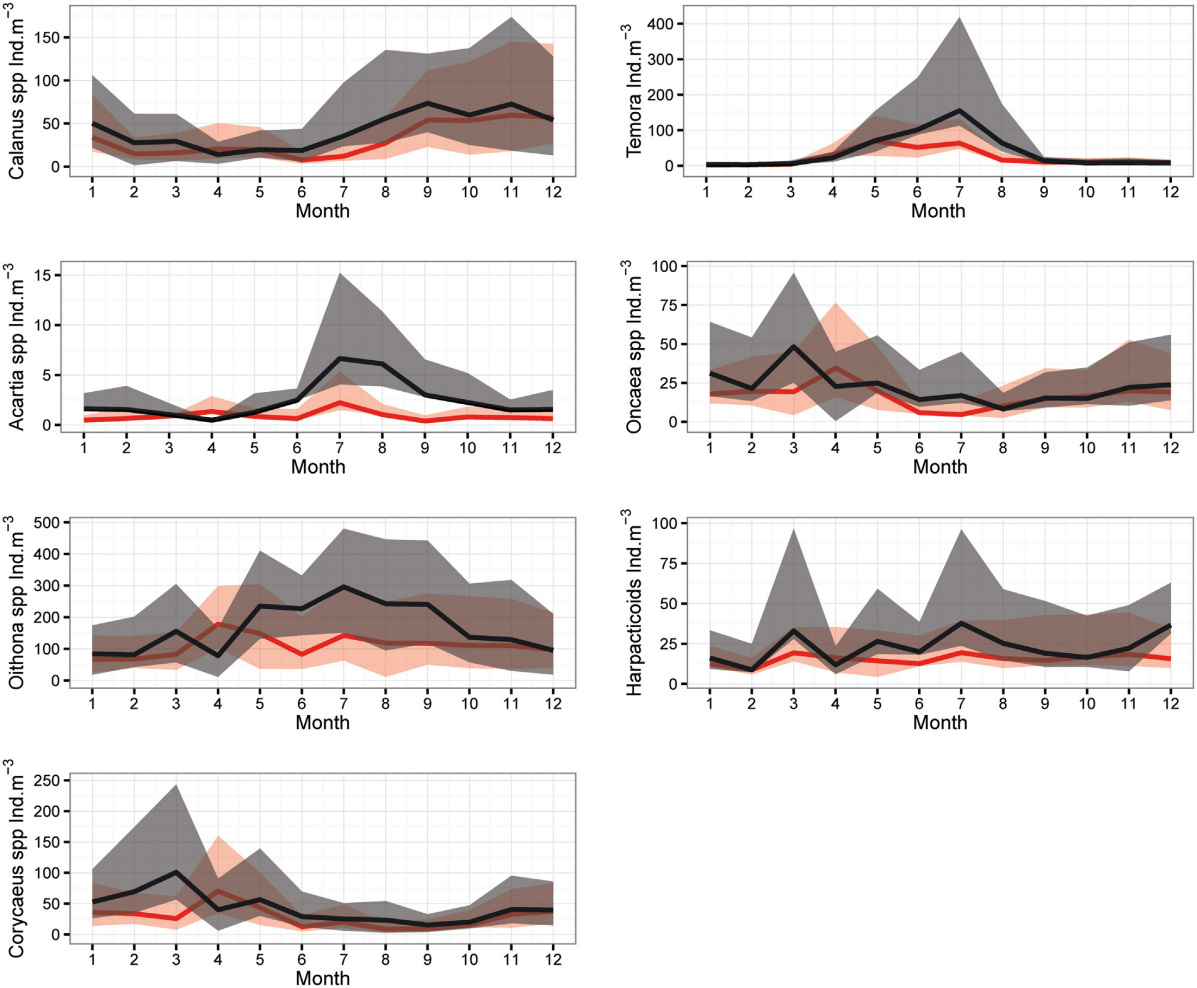
Annual cycle of mean abundances of (A) Calanus spp, (B) Acartia spp, (C) Oithona spp, (D) Corycaeus spp, (E) Temora spp, (F) Oncaea spp and G) Harpacticoïds during SP (black) and NSP (red) with percentiles Q1 and Q3 (shaded area) at B2.

Copepods were the dominant group among the enumerated organisms (~80%, data not shown). Therefore, we chose to present the difference in their annual cycles between the two studied periods (Fig 7). In contrast to the *Temora* spp, *Oncaea* spp and Harpacticoïds (Fig 7E, 7F and 7G, respectively), mean abundances of the *Calanus* spp, *Acartia* spp, *Oithona* spp and *Corycaeus* spp (Fig 7A, 7B, 7C and 7D, respectively) were significantly higher during the SP. An increase was noticed in the mean values of the abundance along the year, except for April where abundances were lower than the NSP. Changes in the timing of the peak have been entailed for some genera. The *Oncaea* spp and *Corycaeus* spp displayed a peak (March) one month earlier in the SP than the NSP (April). While the *Oithona* spp showed a high presence in late spring-early summer thus a peak appeared (July) showing a delay of 2–3 month (April-May).

## Discussion

### The physical long-term changes

Warming has been observed in the Lebanese waters by Abboud-Abi Saab et al. [67] and in the last 30 years by Lakkis [68]. However in the current study, no significant temporal trend was detected in the annual temperature suggesting that warming doesn’t affect point B2 during the studied period. Our results didn’t appear to confirm earlier studies that showed that global warming caused the rise of the sea surface temperature in the EMed. Skliris et al. [69] and Shaltout and Omstedt [70] noticed a positive warming trend of 0.042°C.yr^-1^ in the eastern sub-basin in the last three decades.

The most striking result was the clear salinity increase in the upper 80 m of the water column between 2005 and 2010, from 39.05 to 39.35. The timing of this salinity anomaly was consistent with previous studies in the middle of the 2000s in the upper/intermediate layers in the EMed [38], in the easternmost Levantine basin [40] and in the intermediate and deep layers in the Cretan Sea [39]. This salinity event was related to the changes of the EMed upper thermohaline circulation known as the EMT-*like*; which were exclusively caused by the modification of the deep circulation in the Aegean Sea [37, 38]. These reported periods are very consistent with the identified periods in the present study in terms of timing. Salinity changes likely reflected the presence of the EMT-*like* event. As a result, a new denser DWF pushed the preexisting EMed Dense Water up to the superficial layers favoring the uplift of nutrients. However, this DWF was not dense enough to penetrate into the deep layers of the EMed [37–39]. In 2010, a complete reversal of the Ionian upper layer circulation occurred and the North Ionian Gyre became cyclonic and favored the Adriatic Sea pre-conditioning [39]. Salinity started decreasing and indicated a slow return towards the pre-EMT-*like* conditions. As well, the water mass proportions of the eastern basin had significantly changed between 2008 and 2013 (post-EMT-*like* conditions), and therefore intermediate and deep water mass were noticeably modified [71]. This physical evidence of the change in the hydrology, observed elsewhere, confirmed our choice to define the EMT-*like* being the SP (2005–2010) and non-EMT-*like* period being the NSP (2000–2004 and 2011–2013).

### The plankton temporal evolution

No significant trend was shown on the annual zooplankton abundance over the entire time period. The current time-series may have not been long enough to reveal possible impact of warming. Longer time-series will be required to assess the response of zooplankton to global warming. Instead, our results pointed out to changes in abundances between the two periods related to the occurrence of the EMT-*like*. Total zooplankton abundance significantly increased by 44% during the EMT-*like* period (2005–2010). The abundance of herbivorous and filter feeders (gastropods, pteropods, appendicularia, ostracods, *Evadne* spp, harpacticoids, *Oncaea* spp and *Calanus* spp) increased by a factor of 1.4 (from 96.5 ± 69.8 to 136.4 ± 119.1 ind.m^−3^) matching the rise of carnivorous organisms such as siphonophores, annelids, *Corycaeus* spp, thaliacae and chaetognaths, which increased by a factor of 1.6 (from 75.1 ± 59 to 124.9 ± 107.1 ind.m^−3^). More specifically, siphonophores increased by 110%, annelids 64%, chaetognaths 58%, eggs 50%, copepods 47% and gastropods 19%, being the main groups responsible for this rise. As for copepods, *Acartia* spp, *Oithona* spp, *Corycaeus* spp and *Calanus* spp increased by 212%, 51%, 48% and 35% respectively. For some taxa, differences were almost evident all around the EMT-*like* years. Higher zooplankton abundances and especially copepods also were reported elsewhere in the EMed under saline circumstances subsequent to the EMT onset. For instance, Christou [22] related the increase in total copepod abundances early 1990s in the Aegean Sea to an increase in salinity; which in its turn was related to changes in water mass during the EMT onset. Conversi et al. [42] also reported the increase of annual copepod abundances in the Adriatic Sea and explained this pattern by a change of circulation following the EMT event in the early 1990s. In the Ionian Sea, *Oithona* spp along with *Corycaeus* spp and larger size chaetognaths also became more abundant during the EMT onset [41]. The authors related the changes in chemical characteristics to water mass exchanges and the enrichment effects of the cyclonic circulation. This circulation is influenced by the upward shift of the nutricline to the euphotic zone due to the EMT [36] and the interaction between the cyclonic circulation and the continental slope [41].

A significant increase of phytoplankton abundances (nano and microphytoplankton) was observed during winter-early spring of the EMT-*like* period. They also exhibited a peak earlier than the non EMT-*like* period. Our observations partially agreed with earlier observation off the Israeli coast where higher deep chlorophyll-*a* biomass was observed from 2005 till 2010 [40]. In this latter study, the authors observed an uplift of the nutricline during these years and proposed that it sustained higher phytoplankton biomasses. However, they acknowledged the need for additional measurements in their time-series to confirm the spatial extent of their findings. In contrast to their observations, we observed a significant decrease in nitrate along with a significant increase of orthophosphate (data not shown). However, nutrient concentrations at the site of study may not be solely impacted by the deep waters supply. Their concentrations in the upper 10 m of the surface layer (total nitrites + nitrates and orthophosphates higher than 0.4 μM.L^-1^ and 0.15 μM.L^-1^ respectively, data not shown) are well above reported levels for the oligotrophic Levantine Sea [72]. Kress et al. [40] detected values permanently less than 0.2 μM.L^-1^ for the total nitrites + nitrates and 0.05 μM.L^-1^ for the phosphates in the EMed from 2002 until 2010. Nutrients at B2 probably reflected more the continental inputs from the two major sources (Al-Jaouz river and the chemical industry) that exist in the region [67] than the enhanced vertical mixing during the EMT-*like* period [40]. Coastal morphology, sea state and meteorological conditions can play an essential role in the inputs dispersion and therefore, their effect can be extended far beyond the offshore station [67]. Therefore, in the present study, this nutrient data cannot be used to trace nutrients supply from the deep waters as in Kress et al. [40] and their hypothesis cannot be confirmed neither rejected.

Changes in the phenology were detected in the nanoplankton and in several zooplankton taxa with a predominantly advanced peak during the EMT-*like* period, in addition to other ones later during the same year. It is worth noting that the observed increase of the nanoplankton population during the EMT-*like*, especially in February, probably resulted from enhanced nutrients availability; it was due to the strong mixing as revealed by the weaker stratification at this time of the year during the SP. In addition to the advantageous conditions in the water column, our results suggested an early development of the zooplankton community favored by the earlier production season of phytoplankton. The zooplankton probably showed the preference for a diet based on nanoplanktonic preys. Therefore, the earlier bloom of nanoplankton allowed the early development of the zooplankton communities because of the plasticity in their feeding behavior and habits [41].

We propose, as first hypothesis that the strong mixing during EMT-*like* in the Levantine basin may cause higher primary production which ultimately favored the zooplankton community. However to validate this hypothesis further investigations and supplementary measurements of other ecosystem components (microzooplankton) and rates (primary production, grazing) are required. A second hypothesis that could explain the observed result would be that different zooplankton communities would be conveyed to the study area in the course of changes in surface and deep circulation that altered the Levantine Basin. Weikert et al. [43] reported the occurrence of *Calanus helgolandicus* in deep Levantine Sea due to the intensified water exchange with the Aegean Sea and the upwelled deep water masses during the EMT onset. In the Adriatic Sea, the distribution and the timing of the first appearance of some organisms were considered as an indicator of the EMed water mass entry. For instance, the arrival of *Diaixis pygmoea* in the surface water of the Adriatic Sea during EMT was possibly due to the Ionian Gyre Reversal [42]. In our case, an increase in zooplankton abundance could be partly a result of a water mass transport from more productive regions. Therefore, the observed changes in the abundance could possibly be associated with the circulation changes in the EMed and the water masses modification entering its eastern side. Yet, the detailed procedures were not clear and the experimental evidence supporting this hypothesis is still limited. The second hypothesis will remain enigmatic and should be verified via specific targeted studies.

To conclude, we have presented here the first zooplankton decadal time-series in the Levantine Sea. Salinity has been considered as a good proxy for change in the hydrology following EMT-like events. The interannual changes that took place in the zooplankton community over the whole 2000–2013 period, appeared to be more related and driven by the EMT-*like* dynamics than the sea surface warming. Maintaining this long-term series will provide us a clearer perception on the underlying mechanism influencing zooplankton communities. Trophic interactions triggered by stronger nutrient inputs could explain the observed pattern. Still, transport of different zooplankton community by sea surface circulation cannot be ruled out. This work could be an inducement for further studies to better understand the functioning of the pelagic ecosystem in the Levantine basin. Finally, assessing the consequence of long-term warming on zooplankton requires the continuation of this time-series.

## Supporting Information

S1 Table Hydrological data of Station B2. (CSV)

S2 Table Zooplankton data of Station B2. (CSV)
